# Prognostic Biomarkers Identification in Esophageal Cancer Based on WGCNA and Single-Cell Analysis

**DOI:** 10.1155/2022/6595778

**Published:** 2022-12-16

**Authors:** Wen-jun Zhao, Yao Liu, Jing Guo, Hua Liu

**Affiliations:** ^1^Department of Gastroenterology, The Affiliated Hospital of Qingdao University, Shandong Province, China; ^2^Department of Digestive Endoscopy Center, The Affiliated Hospital of Qingdao University, Shandong Province, China

## Abstract

**Background:**

Esophageal cancer is one of the most common cancers worldwide. Dysregulation of genes plays an important role in cancer. In this study, we aimed to investigate the prognostic biomarkers in esophageal cancer based on comprehensive bioinformatics analysis including WGCNA and single cell analysis.

**Methods:**

RNA sequencing data of esophageal cancer was downloaded from GSE75241 dataset in the GEO database. We also selected esophageal cancer patients from public databases (Genotype-Tissue Expression (GTEx) and The Cancer Genome Atlas (TCGA)). WGCNA was used to construct a scale-free coexpression network of genes. Multifactor Cox analysis model was constructed as the prognostic model in esophageal cancer. Furthermore, single-cell gene analysis was used to discover the mechanism of hub genes in esophageal cancer.

**Results:**

WGCNA discovered 182 genes for further analysis. Among 182 genes, four genes including ANGPT2, VCAN, MS4A4A, and FOS had significant prognostic value in esophageal cancer. In single cell analysis, seven types of cells subsets were distinguished including T cells, B cells, NK cells, monocytes, macrophages, DCs, neutrophils. The expression of four hub genes (ANGPT2, VCAN, MS4A4A, and FOS) in inflammatory cell subsets was evaluated, respectively. Hub genes were correlated with inflammatory cells in esophageal cancer. In addition, the subgroups of specific inflammatory cells such as macrophages, monocytes, and DCs were analyzed to identify the function of hub genes, either. Hub genes were correlated with differentiation of inflammatory cells including monocytes, macrophages, and DCs in tumor environment.

**Conclusions:**

We identified specific hub genes correlated with prognosis of esophageal cancer. These hub genes play critical roles by regulating inflammatory cells status in esophageal cancer.

## 1. Introduction

Esophageal cancer is the eighth most common cancer type and sixth leading cause of cancer deaths cause worldwide [1]. Due to the low 5-year survival rates, esophageal cancer remains a serious problem worldwide. Global statistics show that the incidence of esophageal cancer increases with age, and men are more likely to be diagnosed than women by a ratio of 3 : 1 [2]. Smoking, alcohol, and long-term acid reflux are responsible for the risk factors for esophageal cancer [3]. The symptoms of esophageal cancer are not typical until the cancer has infiltrated over 60%. Management of esophageal cancer includes surgery, chemotherapy, and radiotherapy [4]. Endoscopic mucosal resection could be used in the early stage of the disease. In addition, esophageal cancer is prone to recurrence and metastasis which are correlated with poor prognosis of survival [5]. Thus, it is important to identify the genetic factors to predict the prognosis of esophageal cancer.

In this study, we aimed to discover the hub genes of esophageal cancer by comprehensive bioinformatics analysis. Weighted gene coexpression network analysis (WGCNA) was used to identify key modules and hub genes. In addition, we also used single cell analysis to demonstrate function of hub genes in esophageal cancer. The pseudotime trajectory analysis was used to discover the mechanism of hub genes in inflammatory cell subsets differentiation. This study provides novel insight into the mechanism of esophageal cancer in the future.

## 2. Methods

### 2.1. Data Collection and Data Processing

RNA sequencing data of esophageal cancer were downloaded from GSE75241 dataset in the GEO database (https://www.ncbi.nlm.nih.gov/geo). A total of 30 samples including 15 esophageal cancer tissue samples and 15 normal tissue samples were enrolled in the analysis. The GSE75241 dataset was preprocessed using the “Apy” R package with RMA background correction, Log_2_ transformation, and RNA data normalization. Microarray data probes were converted to gene symbols using the Bioconductor Annotation Data package. We used the GEO 2R online toolkit to screen DEGs between esophageal cancer samples and normal samples. A *P* value <0.05 and a log2-fold change (Fc) >1 were selected as cutoff thresholds. The DEGs were imported into the Metascape database (https://metascape.org/) for functional annotation, respectively [6]. Functional and pathway enrichment analyses were performed with DEGs including KEGG Pathways, and GO Biological Processes. In addition, we selected esophageal cancer patients from public databases (Genotype-Tissue Expression (GTEx) and The Cancer Genome Atlas (TCGA)) (including 653 normal esophageal tissues, 13 adjacent tissues and 182 esophageal cancer tissues) to analysis the survival prognosis and the differential expression of hub genes.

### 2.2. Construction of WGCNA and Functional Enrichment Analysis and Selection of Hub Genes

WGCNA is an algorithm based on high-throughput gene coexpression profiling widely used in the identification of gene coexpression networks in various diseases. In this study, we constructed a scale-free coexpression network of all genes using the WGCNA package in R. Parameters of soft threshold was 20 and truncation block size was 20. Then, we used the average-linkage hierarchical clustering method to cluster genes. Each module in WGCNA was analyzed in the STRING database. The gene significance (GS) and module membership (MM) were calculated. The module with a correlation coefficient of 0.84 and P <0.05 was defined as a hub module. Then, the genes in the hub module were subjected to with Gene Ontology (GO) and KEGG enrichment analysis. The selection criteria for hub genes of important gene modules in WGCNA network are as follows: |GS| >0.6 |MM| >0.6.

### 2.3. Prognostic Model Construction and Clinical Analysis

The hub genes were performed with prognostic analysis. The clinical data of esophageal cancer were downloaded from the TCGA database. The UALCAN database (http://ualcan.path.uab.edu/) was used for the prognostic analysis of the hub gene [7]. The clinical prognostic model was established, and the C-index score of the model was evaluated.

### 2.4. Single-Cell Gene Analysis

The single-cell sequencing data of tumor tissues and adjacent tissues of esophageal cancer were downloaded from the GSE145370 dataset of the GEO database. Raw gene expression matrices were imported and processed using the Seurat v3.0 with default parameters. The cells and genes with poor quality were filtered out. The genes expressed in at least 3 cells and high-quality cells with more than 200 genes and less than 7000 genes were selected for subsequent analysis. Low-quality cells containing more than 20% mitochondrial genes were excluded. Principal component analysis (PCA) with “FindNeighbors” and “FindClusters” functions was used to perform tSNE to screen the significant top 20 principal components (PCs). Then, we clustered the cells with a resolution of dim = 30 and visualized the clustering results using a tSNE scatterplot. Next, the different clusters were annotated to cell types based on the typical marker genes of the cells. Then, the clusters were distinguished into different types of cell populations. Furthermore, we analyzed the expression of hub genes in tumor tissue and adjacent tissue.

The ggplot2 package was used to identify the ratio of cells in tumor with adjacent tissues. For the assessment of the subtypes of specific cell populations, we reanalyzed monocytes, macrophages, and DCs, separately. We used Seurat standard procedures, including PCA dimensionality reduction and tSNE cell construction of clusters to extract cell subsets.

### 2.5. The Pseudotime Trajectory Analysis

In pseudotime trajectory analysis, we reclustered monocytes, macrophages, and DC cells, separately. Seurat standard procedures were used to identify the subsets of each cell type. Monocle (version 2) was applied to determine potential lineage differentiation. First, the top 1000 highly variable genes (HVGs) were selected using the “FindVariableFeatures” function in Seurat v3. Then, we used the “estimateSizeFactors”, “estimateDispersions” and “dispersionTable” functions to build a statistical model to characterize the data. Genes with mean expression >0.1 were retained for subsequent analysis. The “reduceDimension” function of the DDRTree method was used to perform dimension reduction. We also performed branch expression analysis modelling based on the pseudotime analysis. Furthermore, we used the ggplot2 package to identify the ratio of cells in tumor to adjacent tissues. Finally, we identified differentiation of cell subsets based on state, pseudotime, cell type, and group.

### 2.6. Western Blot Analysis

RIPA lysis buffer containing phenylmethylsulfonyl fluoride (PMSF) was used to lysis adjacent normal tissues and tumor tissues of esophageal cancer patients. SDS-PAGE (sodium dodecyl sulfate-polyacrylamide gel electrophoresis) was used to separate the proteins in western blot analysis. Nitrocellulose membranes were used to transfer the proteins. After blocking, the membrane was incubated with primary antibody at 4°C overnight. After incubation, membrane was incubated with secondary antibody for 2 h at room temperature. Enhanced chemiluminescence (ECL) reagent was used to visualize the protein. The angiopoietin-2 (ANGPT2) antibody (CST, 50697S), MS4A4A antibody (CST, 29717S), FosB antibody (CST, 2263S), and *β*-Actin antibody (CST, 4970S) were purchased from Cell Signaling Technology. VCAN antibody (ab177480) and anti-rabbit secondary antibody (ab6721) were purchased from Abcam.

## 3. Results

### 3.1. DEGs Identification and Functional Annotation in Esophageal Cancer

**Figure 1:**
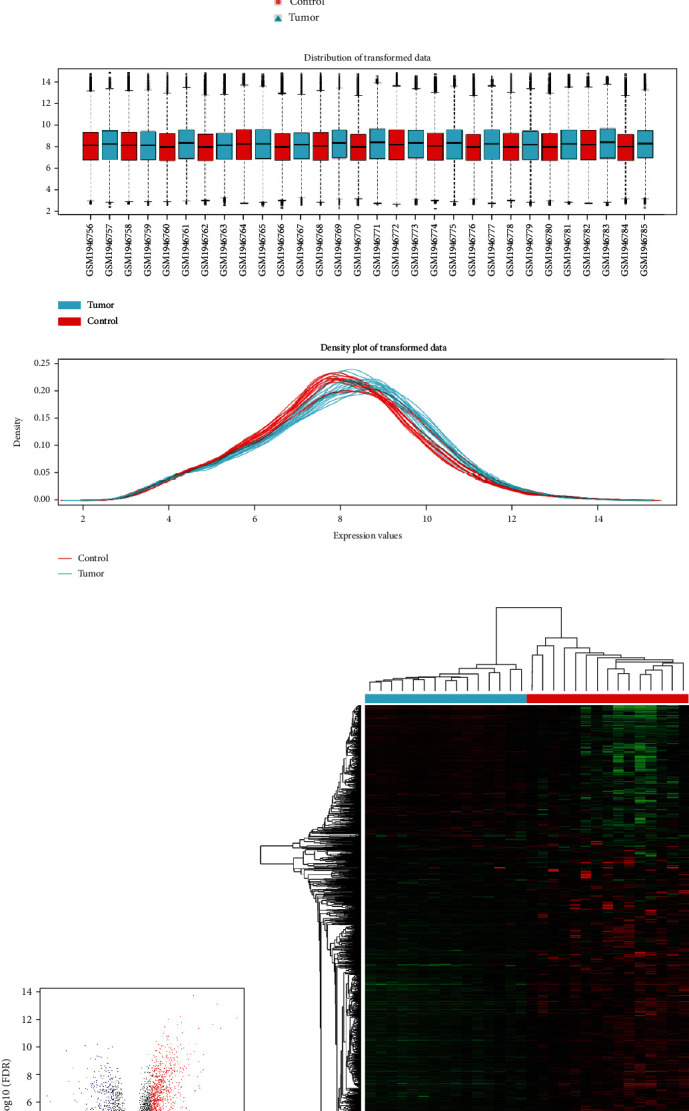
DEGs identification of esophageal cancer. (a) PCA analysis of GSE75241. (b) The box plot of overall distribution of the GSE75241 dataset. (c) The volcano plot of DEGs between tumor tissue and normal tissue. Red dots in the volcano plot represented upregulated genes and blue plots represented downregulated genes. (d) Heat map of DEGs. Green indicated low expression, while red indicated high expression.

We conducted the overall analysis and drew boxplots, transformed data density plots, and principal component analysis (PCA) diagrams (Figure 1(a)). Boxplots showed that the samples were approached with normalization and standardization. Two groups with different samples were obviously distributed in different regions, indicating that the samples were creditable. We identified a total of 1651 DEGs including 1174 upregulated genes and 477 downregulated genes. The volcano plot and heat map of DEGs are shown in Figure 1.

**Figure 2:**
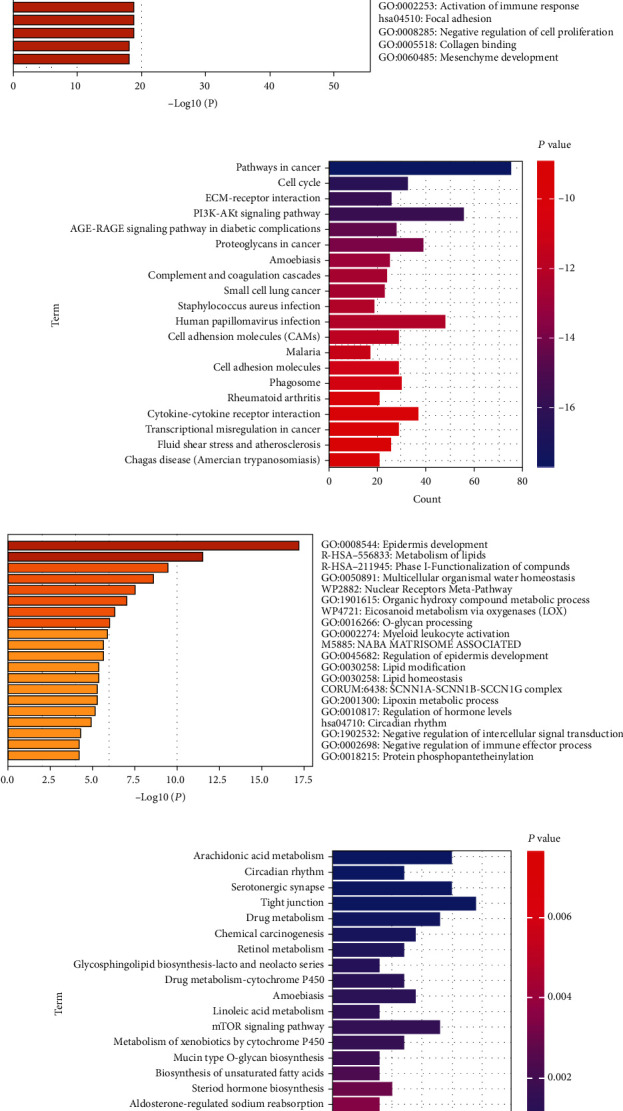
Functional enrichment analysis of DEGs. (a) GO analysis of upregulated DEGs. (b) KEGG analysis of upregulated DEGs. (c) GO analysis of downregulated DEGs. (d) KEGG analysis of downregulated DEGs.

Then, we conducted functional annotation of identified DEGs. In the total 1174 upregulated genes, GO analysis was enriched in extracellular matrix (ECM) organization, regulation of cell adhesion, positive regulation of cell migration, cell division, activation of immune response, tissue morphogenesis, etc. (Figure 2(a)). KEGG analysis of 1174 upregulated genes showed that the pathways were enriched in focal adhesion, the cell cycle, ECM-receptor adhesion, and the PI3K-Akt signaling pathway (Figure 2(b)). In the total 477 downregulated genes, GO analysis was enriched in epidermal development, metabolism of lipids, phase I-functionalization of compounds, etc. (Figure 2(c)). KEGG analysis of 477 downregulated genes showed that the pathways were enriched in arachidonic acid metabolism, serotonergic synapse, drug metabolism, chemical carcinogenesis, etc. (Figure 2(d)).

### 3.2. WGCNA Construction and Key Module Identification and Selection of Hub Gene

**Figure 3:**
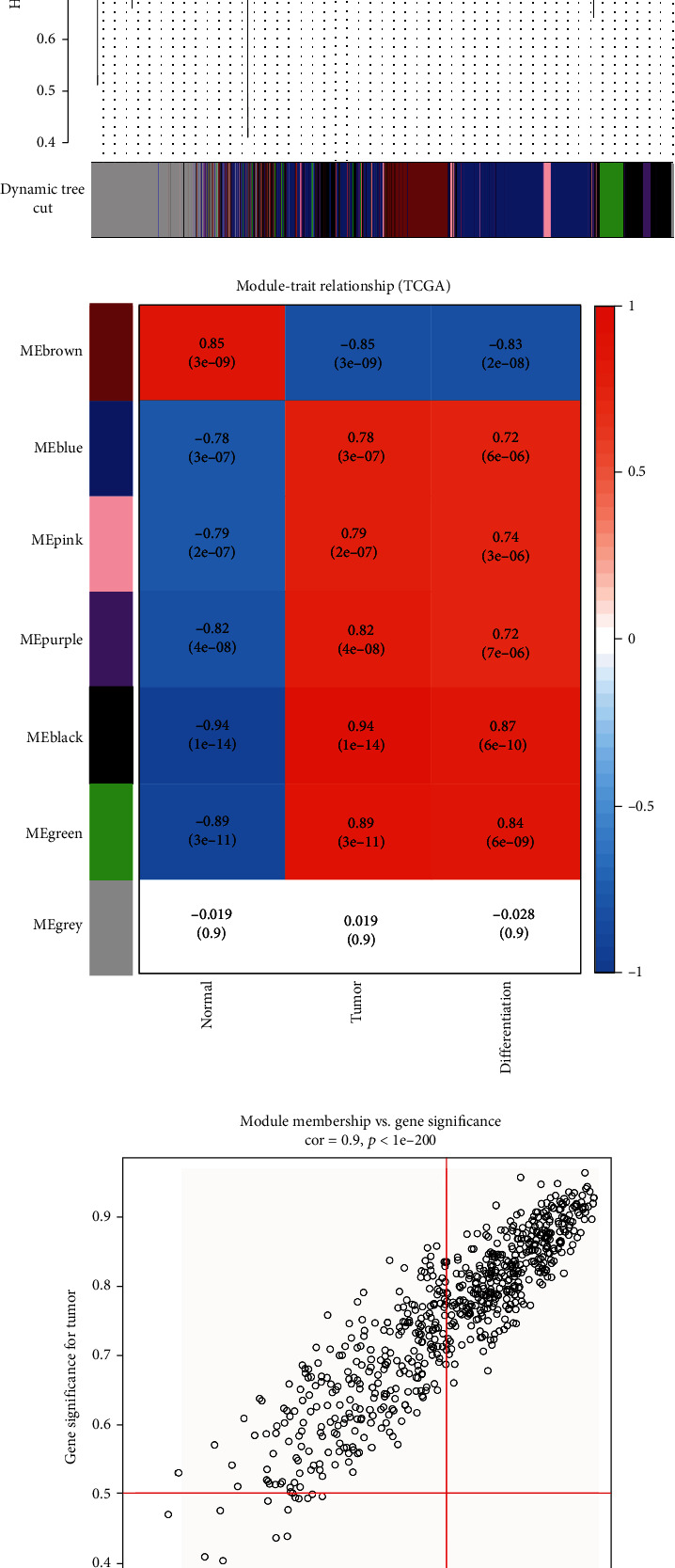
The WGCNA. (a) The clustering dendrogram of tumor and normal samples to detect outliers. (b) Cluster tree of 15 tumor and 30 normal samples in the GSE75241 dataset. The color band underneath the tree indicates the numeric values of the clinical features. (c) The scale-free fit index for soft-thresholding powers (softpower = 20). The soft-thresholding power in the WGCNA was determined based on a scale-free *R*^2^ (*R*^2^ = 0.9). (d) Clustering of module eigengenes. (e) The module-trait relationships between modules and phenotypes. (f) Scatter plot of module eigengenes related to tumor in the black module. (g) Scatter plot of module eigengenes related to tumor in the green module. (h) Scatter plot of module eigengenes related to differentiation in the green module. (i) Scatter plot of module eigengenes related to tumor in the green module.

We used the “WGCNA” package in R to identify key modules. Clustering of samples shows that tumor samples and normal samples are clearly differentiated into two groups. No outliers found in sample clustering (Figures 3(a) and 3(b)). The parameters were listed as follows: soft threshold as *β* = 20 (Figure 3(c)). Five modules were identified with WGCNA package including black module, blue module, brown module, green module, pink module, and grey module (Figure 3(d)). According to the eigenvectors of each module, we calculated the correlation between these modules and phenotype (Figure 3(e)).

Black module and green had high level of correlation with tumor and differentiation degree. According to the expression level of each gene in every sample, we calculated the correlation between the genes and phenotype to measure the gene significance (GS). The larger value of GS had more biological significance, while GS = 0 means that the gene is not related to the phenotype. Black module was correlated with tumor (*P* = 1*e* − 14, correlation = 0.94), green module was correlated with tumor (*P* = 3*e* − 11, correlation = 0.89) (Figures 3(f) and 3(g)). Interesting that Black module was correlated with differentiation (*P* = 6*e* − 10, correlation = 0.87) and green module was correlated with tumor (*P* = 6*e* − 09, correlation = 0.84) (Figures 3(h) and 3(i)). In addition, according to the selection criteria for hub genes of important gene modules in the WGCNA network, we screened 606 genes from the black module and 228 genes from the green module.

**Figure 4:**
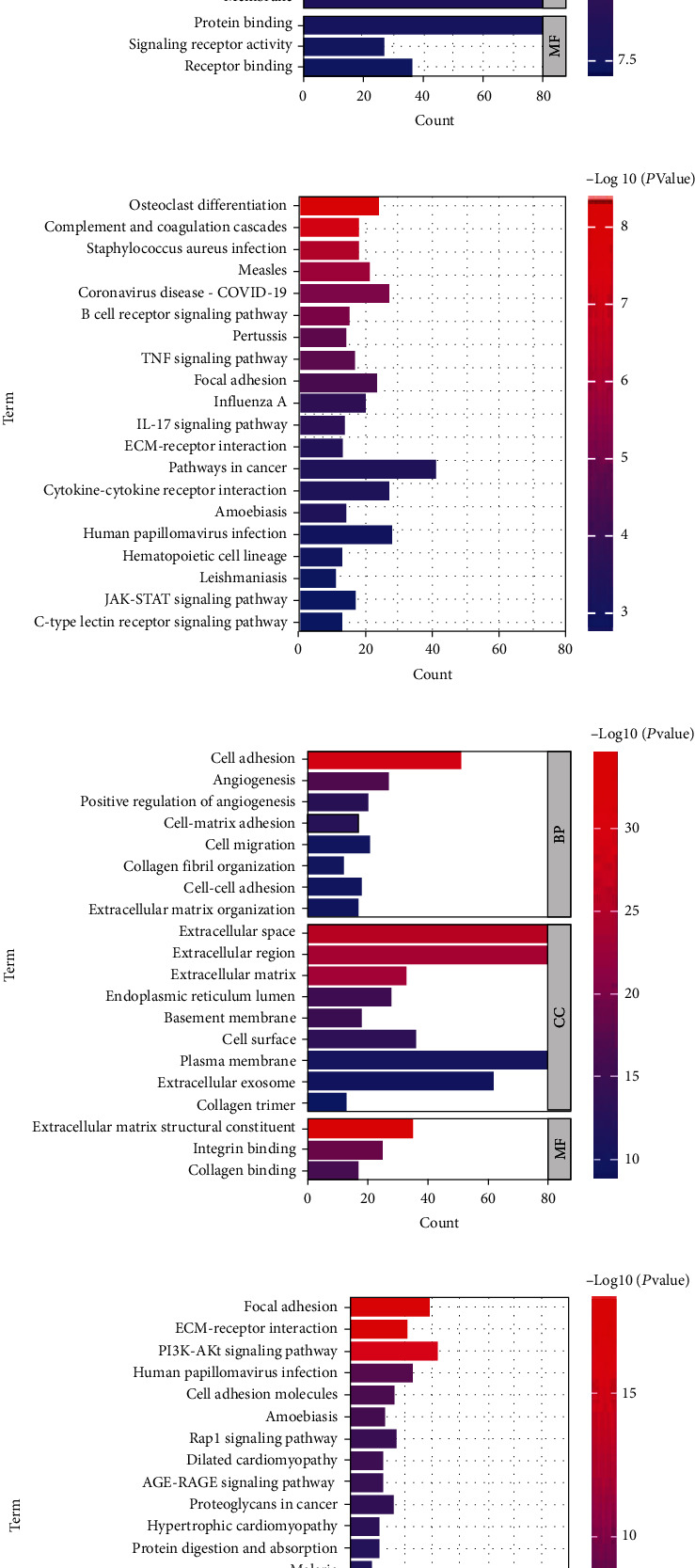
The functional enrichment analysis of turquoise module and blue. (a) The GO analysis of black module. (b) The KEGG analysis of black module. (c) The GO analysis of green modules. (d) The KEGG analysis of green module.

Thus, we selected black and green modules for further analysis. GO analysis of black module showed that the biological processes (BPs) were related with plasma membrane, response to virus, inflammatory response, innate immune response, etc (Figure 4(a)). KEGG analysis of green module showed the pathways were related with complement and coagulation cascades, B cell receptor signaling pathway, IL-17 signaling pathway, Cytokine-cytokine receptor interaction, etc. (Figure 4(b)). GO analysis of green module showed that the BPs were related extracellular matrix structural constituent, cell adhesion, angiogenesis, positive regulation of angiogenesis, cell migration, etc. (Figure 4(c)). KEGG analysis of green module showed the pathways were related with Focal adhesion, ECM-receptor interaction, PI3K-Akt signaling pathway, etc. (Figure 4(d)).

### 3.3. Difference Analysis and Prognostic Analysis of Hub Genes

**Figure 5:**
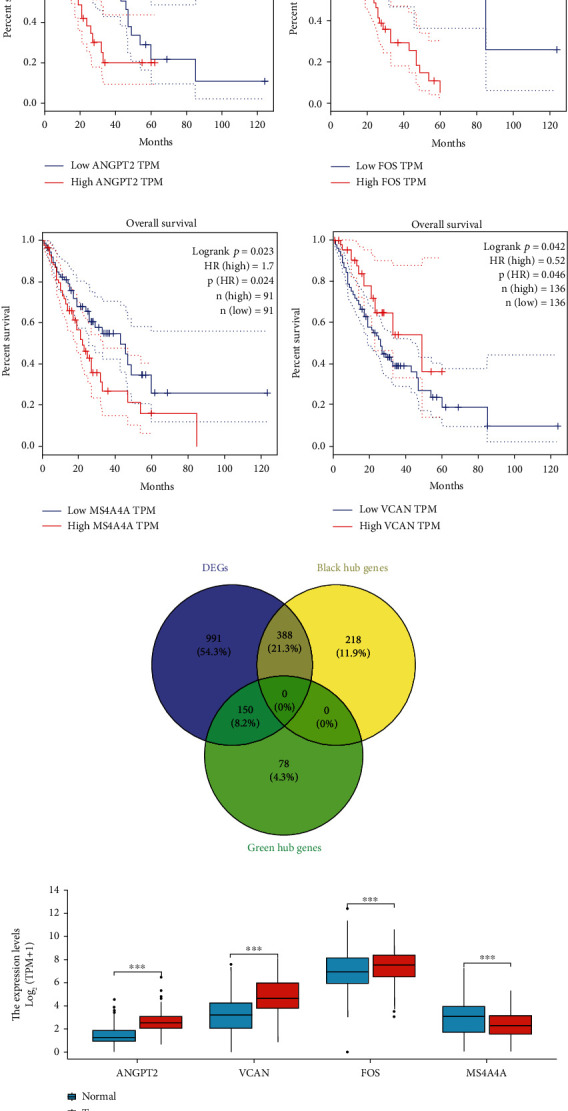
Difference analysis and prognostic analysis of hub genes (ANGPT2, VCAN, MS4A4A, and FOS). (a–d) Prognostic analysis of hub genes (ANGPT2, VCAN, MS4A4A, and FOS) in patients with esophageal cancer. (e) Intersection between genes in important modules of WGCNA and differential genes in normal and tumor tissues. (f) Difference analysis of hub genes (ANGPT2, VCAN, MS4A4A, and FOS) between normal tissues and tumor tissues.

We used the RNA seq. data of esophageal squamous cell carcinoma from TCGA database to analyze the survival prognosis of hub gene. 606 hub genes from the black module and 228 hub genes from the green module obtained from WGCNA. Then we crossed the genes obtained from the important modules of WGCNA with the differential genes between normal tissues and tumor tissues, and finally 388 hub genes from the black module and 150 hub genes are recruited and obtained. These genes are not only differentially expressed genes between normal tissues and tumor tissues but also important genes related to cancer phenotype (Figure 5(e)). Hub genes differentially expressed in normal and cancer tissues were conducted to perform prognostic analysis. The plotted survival curves are shown in Figures 5(a)–5(d). The results indicated four genes including ANGPT2, VCAN, MS4A4A, and FOS had significant prognosis. High levels of ANGPT2, FOS, and MS4A4A were correlated with poor prognosis of esophageal cancer, while high level of VCAN was correlated with better prognosis of esophageal cancer.

We selected esophageal cancer patients from public databases (GTEx and TCGA) (including 653 normal esophageal cancer tissues, 13 adjacent tissues and 182 esophageal cancer tissues). Next, we analyzed the expression of these four important genes (VCAN, ANGPT2, MS4A4A, and FOS) between esophageal cancer and normal esophageal tissues. We found that compared with normal human esophageal tissues, ANGPT2, VCAN, and FOS were significantly upregulated in esophageal cancer tissues, MS4A4A was significantly downregulated in esophageal cancer (Figure 5(f)). Therefore, these four genes may play an important role in the tumorigenesis.

**Figure 6:**
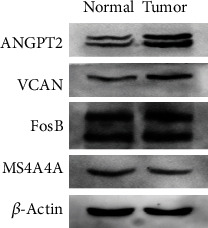
Western blot analysis of ANGPT2, FosB, MS4A4A, and VCAN. ANGPT2, VCAN, and FosB in tumor tissue were higher than normal tissue. MS4A4A in tumor tissue was lower than normal tissue.

Western blot was conducted to evaluate the expression of angiopoietin-2 (ANGPT2), FosB, MS4A4A, and Versican (VCAN) in tumor tissue and normal tissue. The results showed that the expression of ANGPT2, VCAN, and FosB in tumor tissue were higher than normal tissue (Figure 6). The expression of MS4A4A in tumor tissue was lower than normal tissue. The results were in accordance with the difference analysis of hub genes (ANGPT2, VCAN, MS4A4A, and FOS) between tumor tissues and normal tissue.

### 3.4. The Prognostic Model Construction of Esophageal Cancer

**Figure 7:**
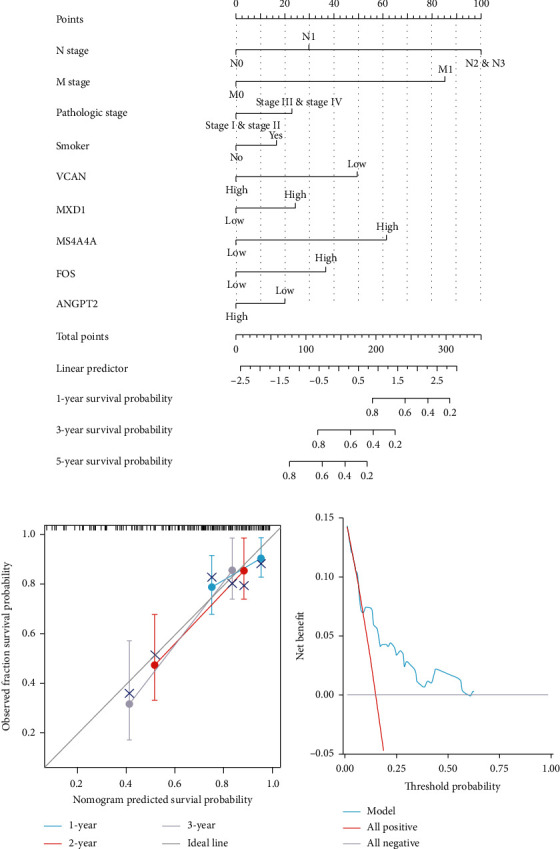
The prognostic model construction of esophageal cancer. (a) Nomogram for predicting 1, 3, and 5-year mortality in patients with esophageal cancer. (b) Calibration curve plots for clinical prediction models. (c) Decision curve analysis plots for clinical prediction models.

We constructed a multifactor Cox analysis model as a prognostic model in esophageal cancer. The results showed that M stage, N stage, VCAN, and MS4A4A are significant predictive factors in multifactor Cox analysis. Based on the results of multifactor Cox analysis, we constructed the nomogram and evaluated the prediction efficiency of the nomogram. The concordance (C-index) was 0.750 (0.695-0.805), indicating that the prediction efficiency of the model was moderately accurate (Figure 7(a)). The prediction model fit well in calibration analysis (Figure 7(b)). The decision curve analysis plot showed that the prediction model could predict the death events of esophageal cancer patients (Figure 7(c)).

### 3.5. Single Cell Analysis of Tumor Tissue and Adjacent Tissue

**Figure 8:**
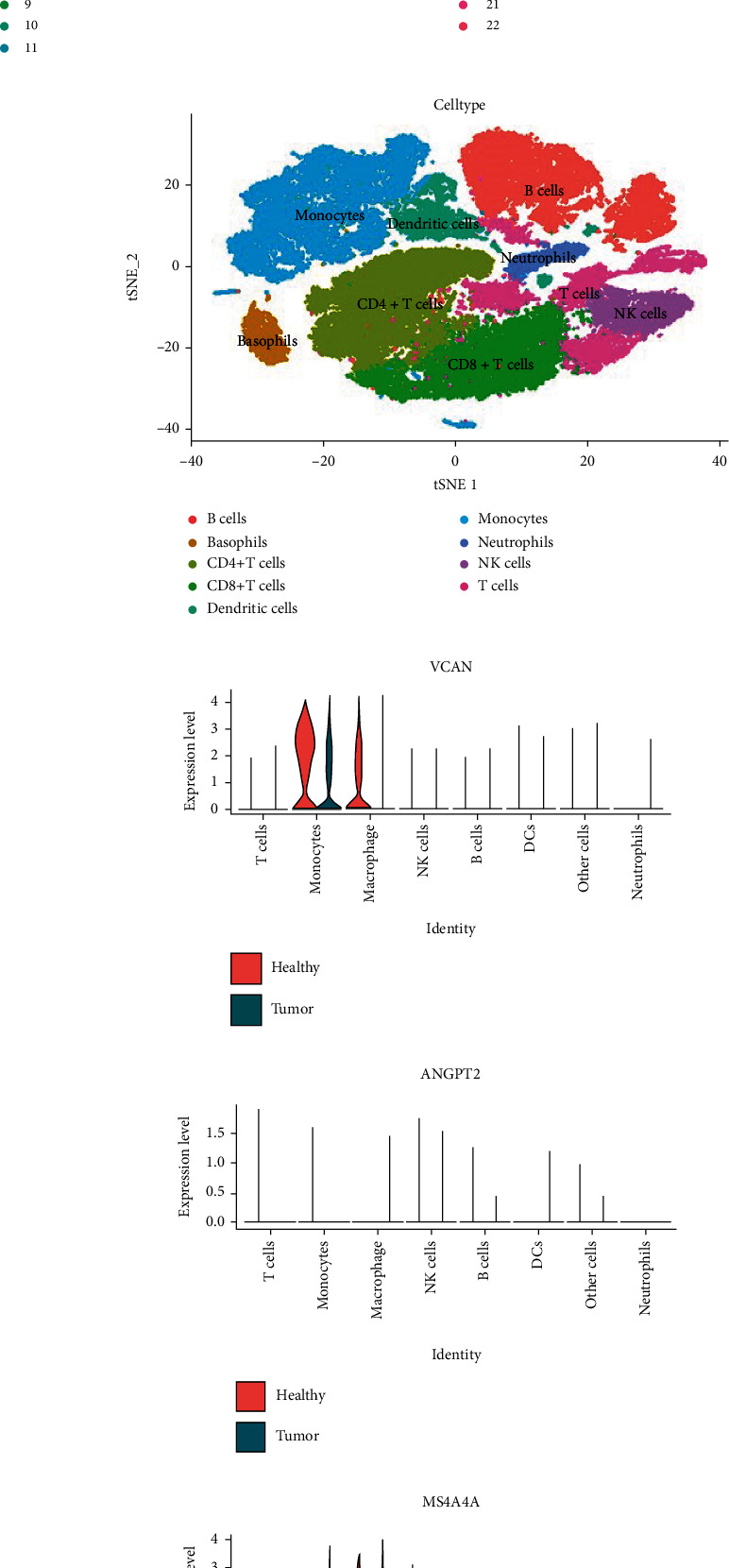
DEGs and cell subsets identification in esophageal cancer with single cell analysis. (a) High quality cell filtration. Cells were filtrated with the number of genes in cells nFeature >200 and ≤7000; the proportion of mitochondrial genes in cells (percent.mt< 20). (b) Filtration of top 2000 highly variable genes (HVGs). (c) Sample pooling and batch correction. (d) The PCA cluster of each sample. (e) The exhibition of cell subsets. (f) The expression of VCAN in tumor tissue and adjacent tissue. (g) The expression of ANGPT2 in tumor tissue and adjacent tissue. (h) The expression of MS4A4A in tumor tissue and adjacent tissue. (i) The expression of FOS in tumor tissue and adjacent tissue. (j) The expression of different immune cell types in tumor tissue and adjacent tissue. (k) The bubble plot of marker gene expression in different cell types identification.

After obtaining hub genes from WGCNA, we conducted single cell analysis to discover the mechanism of hub genes in esophageal cancer. First, we normalized and pooled single-cell data from all samples (Figures 8(a)–8(c)). Then, we merged tumor and adjacent tissue samples to perform unsupervised clustering to identify distinguished cell populations. The cell cluster diagram of different samples showed that there were more NK cells and B cells infiltrating in healthy samples, but more CD4^+^T cells infiltrating in tumor samples (Figure 8(d)). According to the related typical marker genes in immune cells, we classified different immune cell subsets. Seven types of cell subsets were distinguished including T cells, B cells, NK cells, monocytes, macrophages, DCs, and neutrophils (Figure 8(e)). We mainly identified several main types of immune cells such as T cells (CD3D, CD3E, and CD3G), B cells (CD79A, CD79B, and CD19), NK cells (KLRD1, NKG7, and GNLY), monocytes (CD68, LYZ, C1QA, C1QB, and TREM2), macrophages (CD68, LYZ, VCAN, FCN1, and THBS1), dendritic cells (DCs) (CD1C, HLA-DQB2, CD74, and HLA-DQA1), and neutrophils (FCGR3B, MNDA, and ADGRG3). Then, we calculated the expression of four hub genes (ANGPT2, VCAN, MS4A4A, and FOS) in these inflammatory cell subsets. Figures 8(f)–8(i) shows the differential expression of hub genes in cell subsets between tumor samples and adjacent tissue samples. VCAN was highly expressed in macrophages of adjacent tissue samples (P value-adj = 2.02E − 77). In monocytes, VCAN expression was higher in adjacent tissue samples than tumor samples (P value-adj = 9.99E − 84) (Figure 8(f)); ANGPT2 was not highly expressed in immune cells of tumor samples or adjacent tissue samples (Figure 8(g)); MS4A4A was highly expressed in monocytes of tumor samples (*P* value-adj = 1.02E − 14). In macrophages, MS4A4A was more highly expressed in adjacent tissue samples than tumor samples (*P* value  = 0.007) (Figure 8(h)). FOS was highly expressed in all kinds of cell subsets. FOS was more highly expressed in macrophages (P value-adj = 3.98E − 23) and B cells (*P* value-adj = 2.56E − 162) of adjacent tissue samples than tumor samples (Figure 8(i)). In addition, it can be seen that the proportion of neutrophils, DC cells, monocytes, macrophages, and T cells in tumor tissue is upregulated, while the proportion of B cells and NK cells was decreased (Figure 8(j)).  Figure 8(k) shows the marker genes of immune cell subsets.

Furthermore, we analyzed the subgroups of specific inflammatory cells such as macrophages, monocytes, and DCs to identify the function of hub genes.

**Figure 9:**
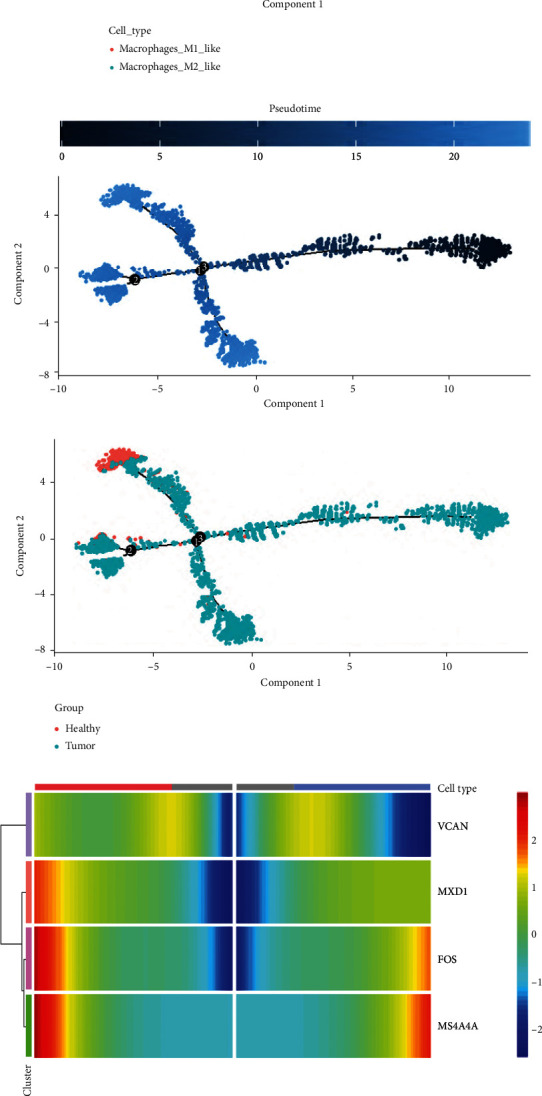
Subpopulation identification and pseudotime trajectory analysis in macrophages. (a) tSNE plot of macrophage cluster. (b) Cell annotation map with marker genes. (c) The differentiation of macrophages in tumor tissue and adjacent tissue. (d) Diagram of pseudotime trajectory differentiation stages of macrophages. (e) Pseudotime trajectory analysis differentiation plot based on cell types. (f) Status of macrophages differentiation by pseudotime trajectory analysis. (g) Pseudotime trajectory differentiation map of macrophages in tumor tissues and adjacent tissue. (h) The heat map of the gene expression during macrophage differentiation. (i) The expression of hub genes during the differentiation of Macrophages_M1_like and Macrophages_M2_like subsets. (j) Macrophages are divided into positive group and negative group according to whether FOS gene is expressed. (k) GO function enrichment results of FOS^+^ Macrophages cells upregulated genes.

Based on marker genes, macrophages could be identified as Macrophages_M1_like (CD14, CCL3, and CD86) and Macrophages_M2_like (CD163, FCGR3A, and C1QC) (Figures 9(a) and 9(b)). In tumor tissue, the expression of Macrophages_M1_like was higher than Macrophages_M2_like. In adjacent tissue, the expression of Macrophages_M1_like was almost the same with Macrophages_M2_like (Figure 9(c)). Through Monocle2 pseudotime trajectory analysis, we showed different stages of macrophage differentiation in esophageal cancer (Figure 9(d)). The performance of each subpopulation in the process of macrophage differentiation is shown in Figure 9(e), and the differentiation with pseudotime is shown in Figure 9(f), the healthy tissue cells are mostly distributed in the 6^th^ and 7^th^ stages, and it can be seen that the Macrophages_M2_like in the healthy tissue Macrophages are massively differentiated into Macrophages_M1_like cells in tumor tissue (Figure 9(g)). In tumor tissue cells, we analyzed the expression of hub genes at different stages of macrophage differentiation. The expression levels of FOS and MS4A4A changed significantly during the macrophage differentiation. The expression of FOS and MS4A4A increased gradually in the macrophage differentiation process (Figures 9(h) and 8(i)). We further analyzed the regulation function of FOS on macrophages. Macrophages are divided into positive group (FOS^+^ Macrophages cells) and negative group (FOS^−^ Macrophages cells) according to where FOS gene is expressed. We analyzed the difference of gene expression between FOS^+^ Macrophages cells and FOS^−^ Macrophages cells (Figure 9(j)). Compared with FOS^−^ Macrophages cells, FOS^+^ Macrophages cells highly express FOS, JUN, JUNB, FOSB, KLF6, etc. We further analyzed the GO function enrichment of the upregulated genes of FOS^+^ Macrophages cells. The upregulated genes are mainly concentrated in the response to lipolysaccharide, positive regulation of cytokine production, and response to molecule of bacterial origin pathway (Figure 9(k)). Therefore, the high expression of FOS is related to the increase of cytokines produced by macrophages and inflammatory reaction, which may also be more similar to the function of M2 macrophages. FOS^+^ Macrophages cells are more likely to promote inflammation and tumor growth.

**Figure 10:**
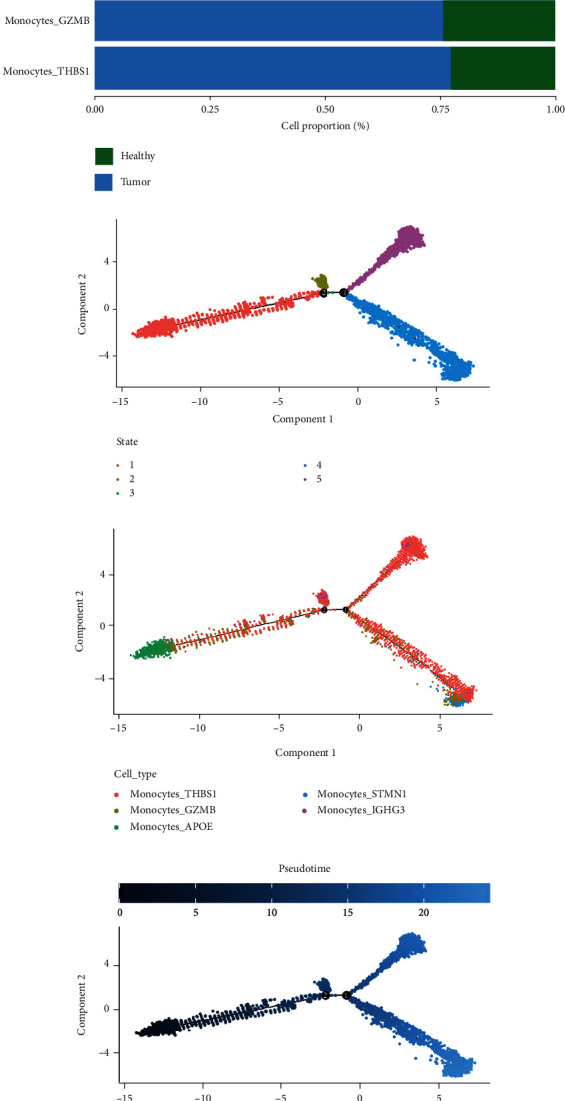
Subpopulation identification and pseudotime trajectory analysis in monocytes. (a) tSNE plot of monocytes cluster. (b) Cell annotation map with marker genes. (c) The differentiation of monocytes in tumor tissue and adjacent tissue. (d) Diagram of pseudotime trajectory differentiation stages of monocytes. (e) Pseudotime trajectory analysis differentiation plot based on cell types. (f) Status of monocytes differentiation by pseudotime trajectory analysis. (g) The heat map of the gene expression during monocyte differentiation. (h) The expression of hub genes during the differentiation of monocyte subsets.

We extracted monocyte subsets and further identified them as Monocytes_THBS1 (S100A8, THBS1), Monocytes_GZMB (S100A8, GZMB), Monocytes_APOE (S100A8, APOE), Monocytes_STMN1 (S100A8, STMN1), and Monocytes_IGHG3 (S100A8, IGHG3) (Figures 10(a) and 10(b)). From the cell ratio, it can be seen that Monocytes_IGHG3 is only present in cancer tissues, Monocytes_APOE is also abundant in tumor tissues, and Monocytes_STMN1 is more distributed in healthy tissues (Figure 10(c)). The pseudotime trajectory analysis of Monocle2 shows that there are 5 differentiation stages of monocytes (Figure 10(d)), and the performance of each subpopulation in the process of monocyte differentiation is shown in Figure 10(e). The result of differentiation according to the pseudotime is shown in Figure 10(f). During the process of monocyte differentiation, the expression changes of the genes we focused on are shown in Figures 10(g) and 10(h). With the progress of cell differentiation, the expression of MS4A4A gene gradually decreased, and the expression of VCAN gene gradually increased.

**Figure 11:**
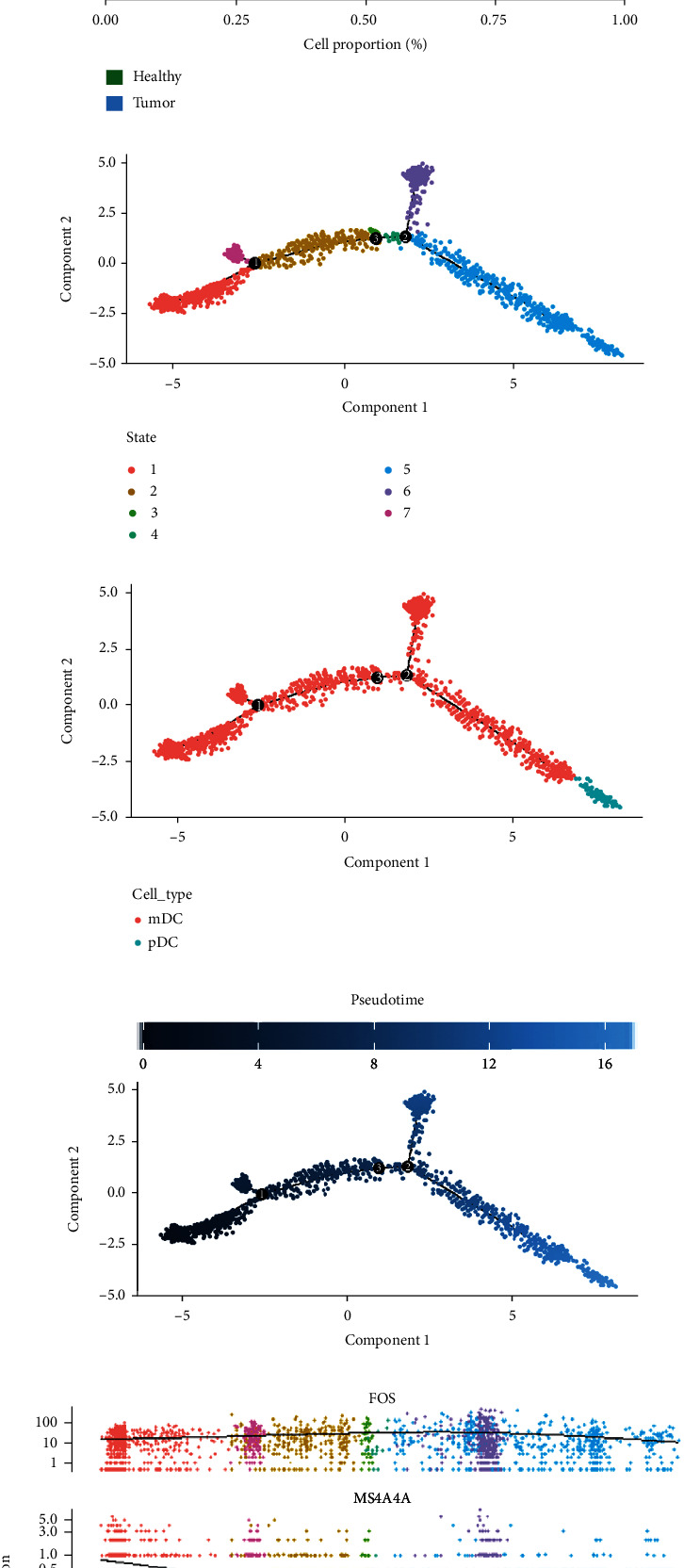
Subpopulation identification and pseudotime trajectory analysis in DCs. (a) tSNE plot of DCs cluster. (b) Cell annotation map with marker genes. (c) The differentiation of DCs in tumor tissue and adjacent tissue. (d) Diagram of pseudotime trajectory differentiation stages of DCs. (e) Pseudotime trajectory analysis differentiation plot based on cell types. (f) Status of DCs differentiation by pseudotime trajectory analysis. (g) The expression of the hub gene expression during DCs differentiation. (h) The heat map of hub genes during the differentiation of DCs subsets.

As for DCs, we extracted DCs cell subsets, and further identified DCs cell subsets as mDCs (LYZ) and pDCs (LILRA4) according to marker genes (Figures 11(a) and 11(b)). It can be seen from the cell ratio that both mDC and pDC are more distributed in tumor tissue, which suggesting that dendritic cells are more active in tumor immunity (Figure 11(c)). Monocle2 pseudotime trajectory analysis revealed that there have 7 differentiation stages of DCs cell differentiation (Figure 11(d)). The performance of mDCs and pDCs during the differentiation process is shown in Figure 11(e), and the differentiation based on the pseudotime is shown in Figure 11(f)). With the cell differentiation, the expression changes of genes we focus on in mDC to pDC cells are shown in Figures 11(g) and 11 (h). MS4A4A gene expression decreased rapidly in the second differentiation stage with the progress of differentiation.

## 4. Discussion

Esophageal cancer is a type of cancer that occurs in the esophagus. The typical symptoms of esophageal cancer are swallowing difficulty and weight loss. Other atypical symptoms of esophageal cancer are hoarse voice, dry cough, and enlarged lymph nodes. Gene dysregulation plays an important role in esophageal cancer. In this study, we attempted to discover the function of prognostic hub genes in esophageal cancer. A total of 182 hub genes were identified from WGCNA. Four genes including ANGPT2, VCAN, MS4A4A, and FOS were considered to have significant prognostic value in esophageal cancer. ANGPT2 belongs to the angiopoietin family of growth factors and expressed in endothelial cells [8]. The protein is the ligand of the endothelial TEK receptor tyrosine kinase. Angiopoietin 2, a growth factor to modulate vessel development, is upregulated in several inflammatory diseases and involved in inflammatory related signaling pathways. In the tumor microenvironment, mesenchymal stem cells can secret ANGPT2. Previous studies found that ANGPT2 was related to cancer-associated microenvironment organization along with metastatic formation [9]. In breast cancer, ANGPT2 was correlated with poor prognosis [10]. ANGPT2 blockade could provide therapeutic benefits of cancer [11]. In this study, we found that ANGPT2 was correlated with poor prognosis in esophageal cancer. VCAN is a member of the aggrecan/versican proteoglycan family. The protein is the major component of the extracellular matrix and is correlated with cell adhesion, proliferation, migration [12]. The protein is produced by several kinds of cells such as endothelial, epithelial, leucocytes. VCAN protein could interact with inflammatory cells involved in autoimmune diseases, cardiovascular and lung disease. In this study, we found that VCAN is related to a better prognosis of esophageal cancer, either. MS4A4A is a member of the membrane-spanning 4A gene family. The encoded protein is characterized in hematopoietic cells and nonlymphoid tissues. Sanyal et al. found that MS4A4A is expressed in the plasma membrane of monocytes. It is considered as a novel cell surface marker for plasma cells and M2 macrophages [13]. MS4A4A can regulate signaling activity of immunoreceptor such as Ig receptors and pattern recognition receptors (PRRs) [14]. In lung inflammation mice, MS4A4A could modulate arginase 1 induction in macrophage polarization [15]. In this study, we found that MS4A4A was a hub gene in esophageal cancer with poor prognosis. FOS gene belongs to Fos gene family including FOS, FOSB, FOSL1, and FOSL2. It could encode leucine zipper protein to involve in cell proliferation, differentiation, and transformation. Previous studies found that the FOS gene is a candidate risk gene for several kinds of cancer such as liver cancer, skin cancer, and osteoblastoma [16–18]. In this study, FOS was discovered as a diagnostic biomarker with poor prognosis in esophageal cancer.

Previous studies have found that tumor was correlated with immune cells. Recent years have revealed that tumor initiation and progression were correlated with monocytes, macrophages, DCs, and neutrophils. These types of cells can regulate tumor microenvironment and interplay with other types of immune cells such as T cells and B cells. In this study, we found that the hub genes were mainly correlated with monocytes, macrophages, DCs, and neutrophils in esophageal cancer. Monocytes can connect innate inflammatory response and adaptive inflammatory response. They can accelerate tumor angiogenesis, and promote immune tolerance. However, monocytes can exert anti-tumor effects by stimulating antigen presenting cells (APCs) [19]. In the tumor microenvironment, monocytes can derivate to tumor-associated macrophages (TAMs), tumor-associated DCs, and myeloid derived suppressor cells [20]. Environmental stress, pathophysiological conditions, and cell signals could alter monocyte differentiation in the tumor microenvironment [21]. In this study, we found that the hub genes including VCAN MS4A4A, and FOS could regulate the differentiation of monocytes in the tumor environment. The pseudotime trajectory analysis in single cells showed that specific hub gene was expressed in different subsets of monocytes. Thus, these hub genes might exert antitumor or promote tumor effects by regulating monocytes.

We also found that MS4A4A VCAN, and FOS were correlated with macrophages. Macrophages are an important factor in tumorigenesis and tumor metastasis in tumor microenvironment [22]. Macrophages can influence tumor cell invasion, migration, and metastasis. Several aspects of mechanisms were involved in the functions of macrophages in tumor. Macrophages can promote proliferation of tumor by secreting growth factors including epidermal growth factor receptor (EGFR) [23]. In metastasis and angiogenesis, macrophages can disintegrate the extracellular matrix and regulate the production of immunosuppressive cytokines [24]. Macrophages can also influence immunosuppression in tumor microenvironment [25]. Previous studies have found that the subsets of macrophages including M1 and M2 macrophages play different functions in tumor. M1 exerted antitumor effects, while M2 exerted as protumor effects. M1 macrophages can produce and secret inflammatory factors including nitric reactive oxygen species, TNF-*α*, and nitric oxide. These inflammatory factors can activate antitumor T cell response to kill tumor cells [26]. M2 macrophages can stimulate tumor growth angiogenesis, metastasis by secreting a variety of inflammatory factors such as TGF-*β*, platelet-derived growth factor, and epidermal growth factor [27]. In this study, we discovered that the hub genes in esophageal cancer could influence the differentiation of macrophages from M1 to M2. The expression of FOS, MS4A4A was upregulated in the macrophage differentiation. These genes were correlated with poor prognosis of esophageal cancer. Thus, FOS and MS4A4A might participate in the function of macrophages in esophageal cancer.

DCs are important factors in innate and adaptive immune responses. There is increasing knowledge of DCs in tumor immune responses [28]. DCs act as antigen processing cells in tumor microenvironment to activate naive T cells. The priming and migration of T cells were determined by DCs. In addition, DCs can also generate the tertiary lymphoid structures in tumor microenvironment [29]. DCs were correlated with immunity in radiotherapy, chemotherapy, and checkpoint blockade. In this study, we found that the hub genes were related to DCs in esophageal cancer. MS4A4A was related with the differentiation of DCs. Thus, MS4A4A might influence the progression of esophageal cancer by regulating DCs function.

As a member of innate immunity, neutrophils play an important role in tumor. In recent years, research has discovered that neutrophils were correlated with tumor angiogenesis, growth, and extracellular matrix remodeling [30]. In the origin of cancer, neutrophils influence the activity of tissue stem cells [31]. Regarding angiogenesis, neutrophils can exert angiogenic effects by secreting angiogenic factors. NETosis in tumor microenvironment contributes to tumor metastasis [32]. Neutrophils can also modulate the macrophage status in tumor microenvironment [33]. We found that VCAN and MS4A4A expression was higher in tumor tissue compared with adjacent tissue of Neutrophils. Thus, VCAN and MS4A4A also participated the effect of neutrophils in esophageal cancer.

## 5. Conclusion

In conclusion, we used comprehensive bioinformatics analysis including WGCNA and single cell analysis to discover the prognostic biomarkers of esophageal cancer. A total of 1651 DEGs including 1174 up-regulated genes and 477 down-regulated genes were identified in esophageal cancer. In WGCNA, 182 genes were identified. In the total 182 hub genes, four hub genes including ANGPT2, VCAN, MS4A4A, and FOS had significant prognostic value in esophageal cancer. High levels of ANGPT2, FOS, and MS4A4A were correlated with poor prognosis of esophageal cancer, while high levels of VCAN were correlated with better prognosis of esophageal cancer. In the prognostic model construction of esophageal cancer, multifactor Cox analysis showed that M stage, N stage, VCAN, and MS4A4A were significant predictive factors of esophageal cancer and the model prediction efficiency was moderate accurate. Furthermore, we conducted single cell analysis to discover the mechanism of hub genes in esophageal cancer. Seven types of cell subsets were distinguished including T cells, B cells, NK cells, monocytes, macrophages, DCs, and neutrophils. The expression of four hub genes (ANGPT2, VCAN, MS4A4A, and FOS) in these inflammatory cell subsets was evaluated. Finally, we analyzed the subgroups of specific inflammatory cells such as macrophages, monocytes, and DCs to identify the functions of hub genes. FOS and MS4A4A were correlated with macrophage differentiation process. The expression of FOS and MS4A4A increased gradually in macrophage differentiation process. MS4A4A and VCAN were correlated with process of monocyte differentiation. The expression of MS4A4A gene gradually decreased, and the expression of VCAN gene gradually increased in the monocyte differentiation. MS4A4A was correlated with DCs differentiation. MS4A4A gene expression decreased rapidly in the second stage of DCs differentiation. Thus, we considered that hub genes including ANGPT2, VCAN, MS4A4A, and FOS were involved in the pathogenesis of esophageal cancer. These hub genes changed the inflammatory status in esophageal cancer.

## Data Availability

The data that support the findings of this study are available from the corresponding author.
